# Supplementary material for: Ageratina adenophora causes spleen toxicity by inducing oxidative stress and pyroptosis in mice
Source: R Soc Open Sci. 2019 Jul 24;6(7):190127. doi: 10.1098/rsos.190127 (PMC6689578; doi:10.1098/rsos.190127)
Supplement: Table S1; Figure S.1; Figure S.2 [file rsos190127supp1.docx]

**Supplementary**

Table S1. The primers sequences used for qRT-PCR

| Primer names | Sense sequence(5’-3’) | Antisense sequence(5’-3’) | Product size |
| --- | --- | --- | --- |
| CAT | AGGTGTTGAACGAGGAGGAGAGG | AGCGTTGTACTTGTCCAGAAGAGC | 161 |
| Mn-SOD | ACGCCACCGAGGAGAAGTACC | GCTTGATAGCCTCCAGCAACTCTC | 180 |
| CuZn-SOD | AAGCGGTGTGCGTGCTGAAG | TCCTGACAACACAACTGGTTCACC | 92 |
| GSH-Px | CACAGTCCACCGTGTATGCCTTC | ACCGAGCACCACCAGTCCAC | 191 |
| IL-1β | TCGCAGCAGCACATCAACAAGAG | TGCTCATGTCCTCATCCTGGAAGG | 118 |
| GSDMD | ACTGAGGTCCACAGCCAAGAGG | GCCACTCGGAATGCCAGGATG | 140 |
| Caspase-1 | ACAACCACTCGTACACGTCTTGC | CCAGATCCTCCAGCAGCAACTTC | 118 |
| NF-κB | ATCCACCTCACCGGCCTCATC | TTCGCTGGCTAATGGCTTGCTC | 162 |
| NLRP3 | GAGCTGGACCTCAGTGACAATGC | ACCAATGCGAGATCCTGACAACAC | 146 |
| β-actin | CCTAGGCACCAGGGTGTGAT | TCCATGTCGTCCCAGTTGGT | 137 |


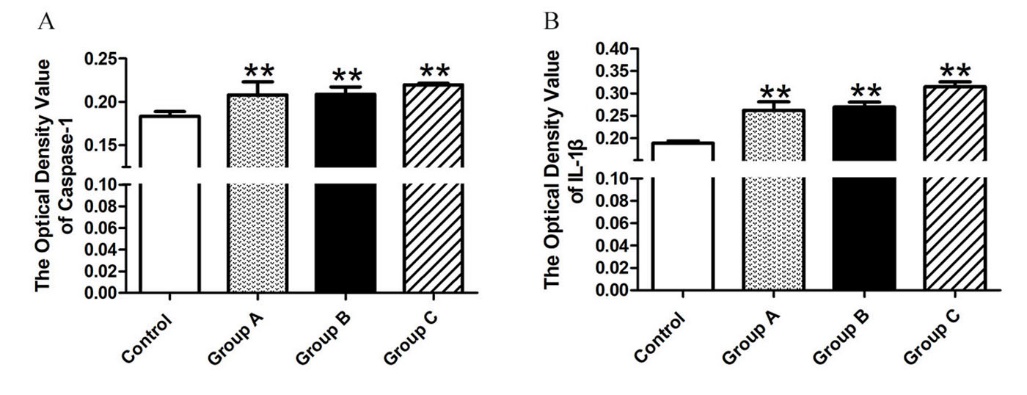


Figure S.1 The change of optical value of caspase-1 and IL-1β measured by immunohistochemistry. (A). The change of caspase-1 optical value in all experiments. (B) The change of IL-1β optical value in control group and *A. adenophora* administration. The optical density values were measured by three random microscopes.


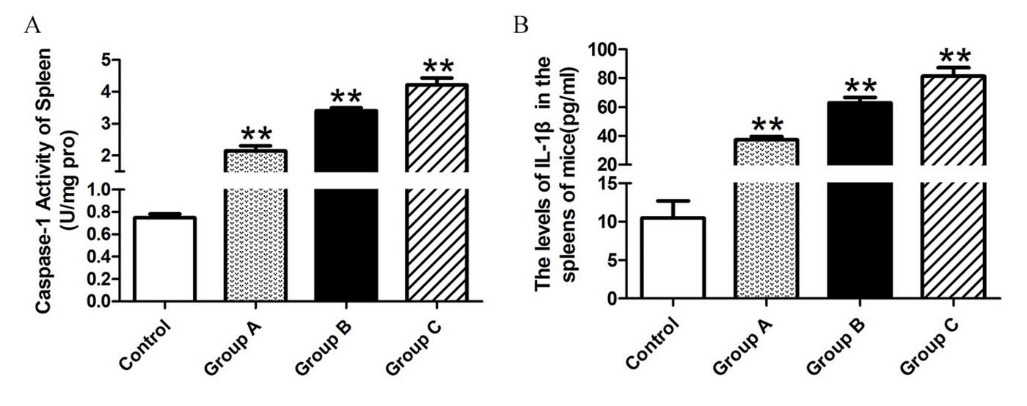


**Figure S.2.** The change of caspase-1 activity and IL-1β levels induced by A. adenophora. (A) The activity of caspase-1 increased with the increasing dosage of A. adenophora. (B) An increasing tendency of IL-1β level was observed in group A, B and C compared with the control group.
